# Laparoscopic Navigated Liver Resection: Technical Aspects and Clinical Practice in Benign Liver Tumors

**DOI:** 10.1155/2012/265918

**Published:** 2012-10-22

**Authors:** Markus Kleemann, Steffen Deichmann, Hamed Esnaashari, Armin Besirevic, Osama Shahin, Hans-Peter Bruch, Tilman Laubert

**Affiliations:** ^1^Department of Surgery, University Hospital Schleswig-Holstein, Campus Luebeck, Ratzeburger Allee 160, 23538 Luebeck, Germany; ^2^Institute for Robotics and Cognitive Systems, University of Luebeck, Ratzeburger Allee 160, 23538 Luebeck, Germany; ^3^Graduate School for Computing in Medicine and Life Sciences, University of Luebeck, Ratzeburger Allee 160, 23538 Luebeck, Germany

## Abstract

Laparoscopic liver resection has been performed mostly in centers with an extended expertise in both hepatobiliary and laparoscopic surgery and only in highly selected patients. In order to overcome the obstacles of this technique through improved intraoperative visualization we developed a laparoscopic navigation system (LapAssistent) to register pre-operatively reconstructed three-dimensional CT or MRI scans within the intra-operative field. After experimental development of the navigation system, we commenced with the clinical use of navigation-assisted laparoscopic liver surgery in January 2010. In this paper we report the technical aspects of the navigation system and the clinical use in one patient with a large benign adenoma. Preoperative planning data were calculated by Fraunhofer MeVis Bremen, Germany. After calibration of the system including camera, laparoscopic instruments, and the intraoperative ultrasound scanner we registered the surface of the liver. Applying the navigated ultrasound the preoperatively planned resection plane was then overlain with the patient's liver. The laparoscopic navigation system could be used under sterile conditions and it was possible to register and visualize the preoperatively planned resection plane. These first results now have to be validated and certified in a larger patient collective. A nationwide prospective multicenter study (ProNavic I) has been conducted and launched.

## 1. Introduction

Laparoscopic liver surgery has been performed throughout the last 20 years. Fabiani et al. first described the minimally invasive fenestration of large solitary liver cysts in 1991 [1]. Four years later, Gagner et al. performed the first laparoscopic wedge resection in a patient with focal nodular hyperplasia. The first oncologic resection was an atypical laparoscopic resection of a liver metastasis by Wayand and Woisetschlager reported in 1993 [2] and in 1996 Azagra et al. reported the first laparoscopic resection of the segments II and III [3]. In 2000, the first larger prospective cohort study on laparoscopic liver resections was published by Cherqui et al. [4]. A multicenter study reported by Gigot et al. 2002 showed the feasibility, safety, and patient outcome of laparoscopic liver resections [5]. Small tumors in the left lateral (II, III) and the anterior segments IVb, V, and VI were found suitable for the laparoscopic approach. Since that time, a number of centers reported larger series including major liver resections, for example, right hepatectomies and right liver lobe resection for living donor transplantation [6]. However, major laparoscopic resections are still discussed controversially. A German survey revealed that about 70% of laparoscopic resections are atypical (wedge) resections. Major liver resections are still rarely performed in Germany [7]. A few groups reported laparoscopic hemihepatectomies with control of the pedicle and even the intrahepatic Glisson approach has been described [8, 9]. Several case reports have shown the technical feasibility of laparoscopic central resections (Mesohepatectomy) [10]. These surgical procedures are highly ambitious and certainly require profound experience in hepatobiliary and laparoscopic surgery.

The first navigation system for laparoscopic surgery in the abdomen was presented by Ellsmere et al. [11]. The goal of this system was not to support hepatic surgery, but rather to improve the orientation in the use of laparoscopic (2D) ultrasound by visualization of the ultrasonic level in relation to the aorta and the large abdominal arteries. The preoperative volume data were recorded using a landmark-based method for the intraoperative situation. CustusX (SINTEF, Norway) constitutes another navigation system for laparoscopic surgery [12]. A devised laparoscopic pointer helps to control the visualization and provides individual images from the preoperative data. The tracking of the instruments is carried out by an optical system. It does not use intraoperative imaging. Instead, preoperative CT/MRI images are registered on a landmark-based method projected onto skin markers. Another system working without any preoperative planning data was constructed by Osaka and Kyushu University, Japan. By tracking a flexible laparoscopic ultrasound probe, a 3D-ultrasound volume is generated [13]. The segmented vascular structures and tumors from the 3D-ultrasound data are visualized and augmented into the laparoscopic video. In the general context of navigated laparoscopy, the compensation of the respiratory movements and the support of laparoscopes with angled optics have to be emphasized [14, 15]. Feuerstein et al. developed a navigation system without the use of preoperative data. The vessel architecture was visualized by laparoscopic ultrasound or radiologic recordings and was fused with the laparoscopic image. 

In order to compensate the limited tactility in laparoscopic liver resection and to use guidance by preoperative resection planning data, we developed a laparoscopic navigation system (LapAssistent). It enables the intraoperative presentation of preoperative 3D-image data of the liver, including intrahepatic vessels, tumor location, and estimated residual functional liver volume. The technical aspects of the LapAssistent system are as follows: (1) presentation of all views provided by MeVis planning, (2) no visibility problem in tracking, (3) preoperative calibration, (4) preregistration manually or by landmark selection, (5) documentation of intraoperative tumor location, (6) online update of resection planning, and (7) navigation guidance for laparoscopic tumor ablation.

In this paper we describe the LapAssistent system and exemplarily report the navigated laparoscopic liver resection in a patient with benign liver cell adenoma in segment VI. 

## 2. Materials and Methods

### 2.1. Patient

Due to an uncertain raise in alcalic phosphatase and unspecific pain in the right upper abdomen a 28-year-old female patient (170 cm, 58 kg) underwent further diagnostic exploration. The abdominal ultrasound revealed a lesion of low echogenicity in the right upper abdominal quadrant with relation to the liver. A subsequent MRI scan showed an oval lesion at the right lower lobe of the liver measuring 5 × 9, 5 cm in craniocaudal extension. According to the imaging there were no signs of an infiltration of healthy liver tissue. Also, there were no signs of pathologically enlarged lymph nodes or ascitis. The preliminary diagnosis was a liver cell adenoma.

### 2.2. Preoperative Preparation of MRI Data

**Figure 1:**
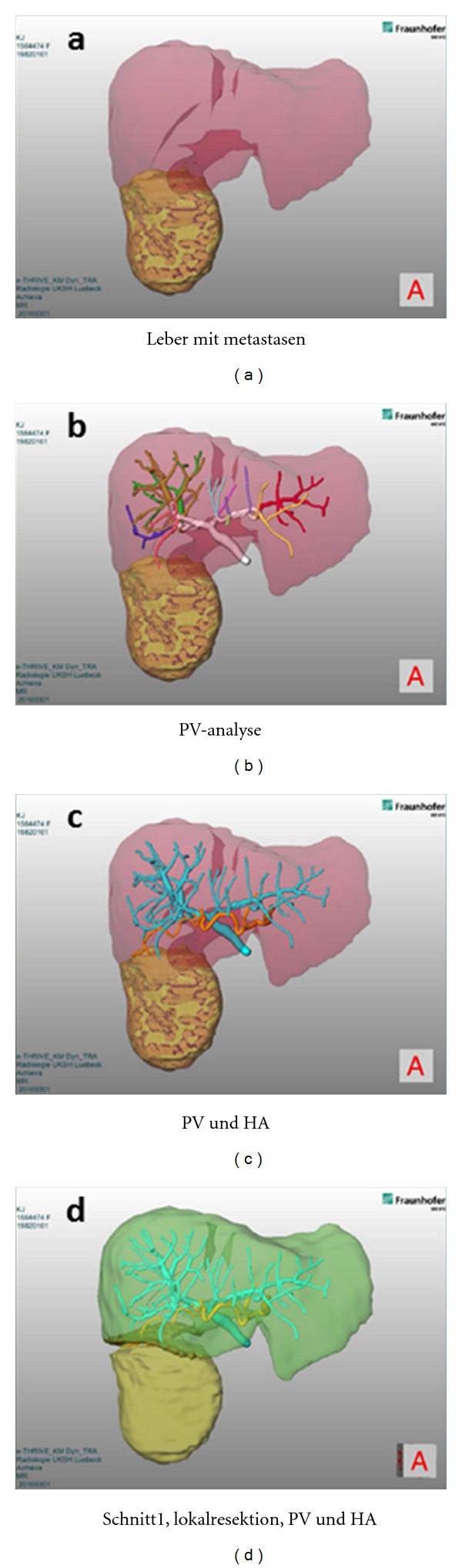
3D MeVis data. (a) liver anatomy and tumor, (b) liver anatomy and portal veins, (c) liver anatomy, portal veins, and hepatic arteries, (d) potential resection plane.

The preoperatively generated MRI-data of the abdominal scan were provided to the cooperation partner MeVis (MDS, Fraunhofer) in Bremen, Germany, for additional calculation and generation of three-dimensional data reconstruction (Figure 1). Besides the individual anatomy of the liver the 3D-reconstruction depicts tumors and vascular structures and allows to interactively explore the patient's liver. Also, volumes of resected and remaining organ areas are calculated and shown in the model. Based on this data, suggestions for potential resection planes have been made. 

### 2.3. Technical Aspects of the LapAssistent

**Figure 2:**
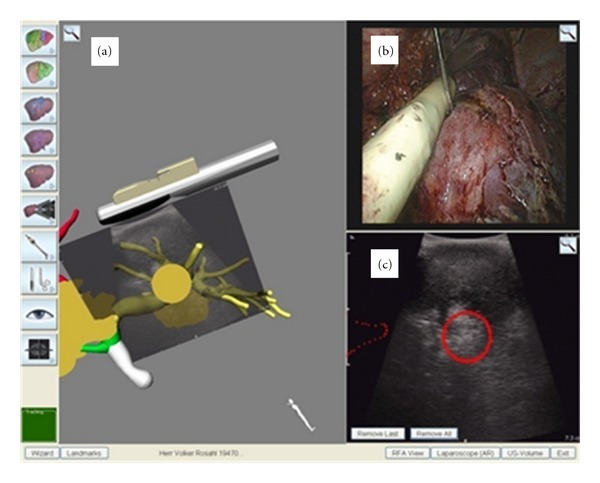
(a–c) Screen shot of the LapAssistent with simultaneous presentation of (a) virtual reality navigation with registered intraoperative B-Mode ultrasound picture to the preoperative 3D-MRI-planning data, (b) real-time laparoscopic video screen imported from surgical laparoscopic imaging device, and (c) real-time ultrasound B-Mode picture with overlapping tumor.

The hardware components of the LapAssistent consist of a touch-screen monitor, a SpaceMouse, a keyboard, and a standard PC (Shuttle PC, 4GB RAM, Intel Core II Duo 6600, GeForce 7950 GX2). The essential part of the system is the electromagnetic tracking device (3D Guidance, Ascension, Burlington, USA) with connection plugs, tracking cable, transmitter, interface and a rack. The components of the navigation software consist of a main window with the following applications:

- virtual reality navigation (Figure 2(a)),
- laparoscopic screen (Figure 2(b)),
- real-time ultrasound picture (Figure 2(c)),
- menu for changing views,
- menu with general applications such as the setting of landmarks and starting of wizards.

### 2.4. Intraoperative Calibration

**Figure 3:**
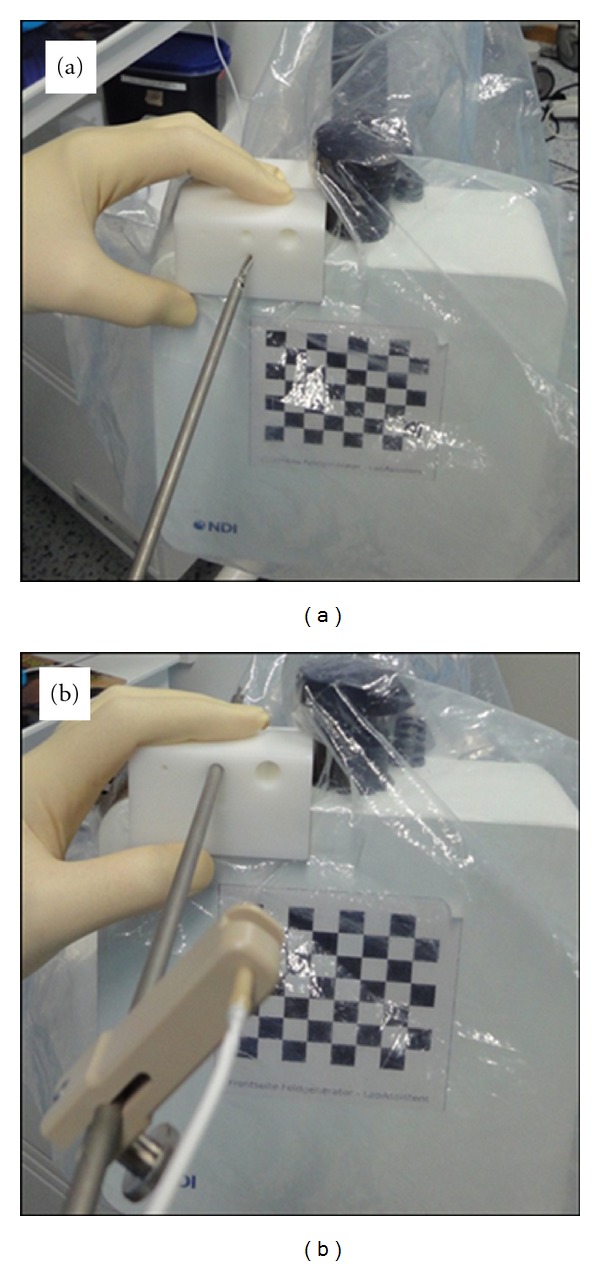
System calibration under sterile conditions. (a) Pivot shift calibration, (b) rotating calibration.

**Figure 4:**
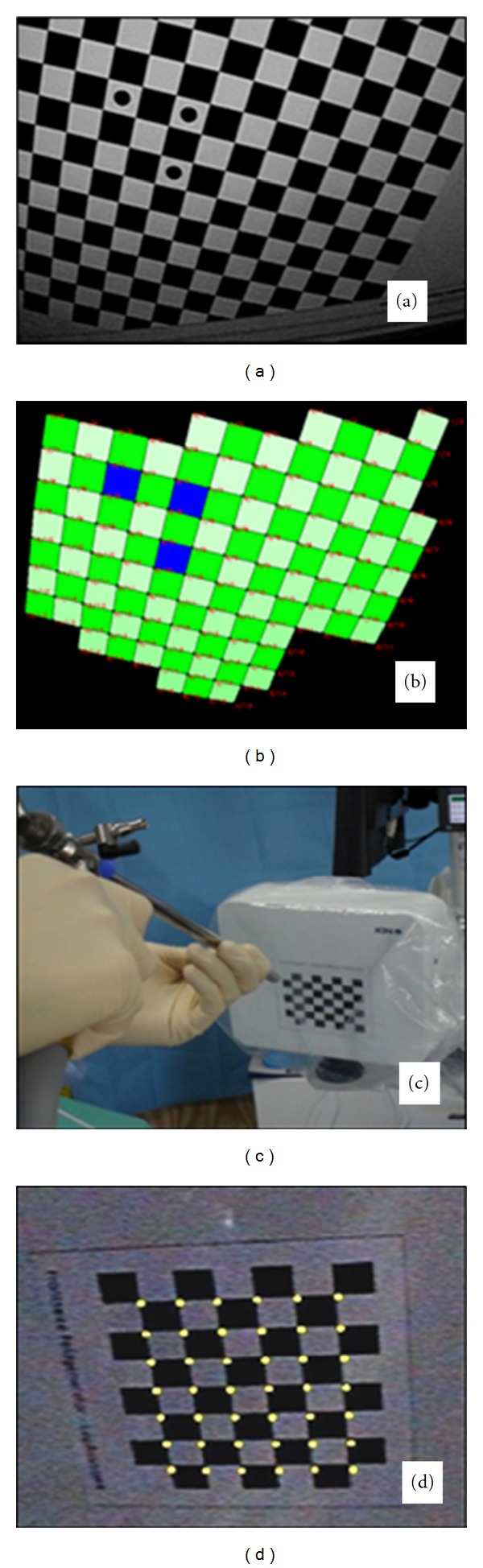
Calibration of the camera. (a) Monitor picture, (b) virtually generated squares, (c) exposure of the squares to the camera, (d) calibrated camera view by definition of square crossings.

**Figure 5:**
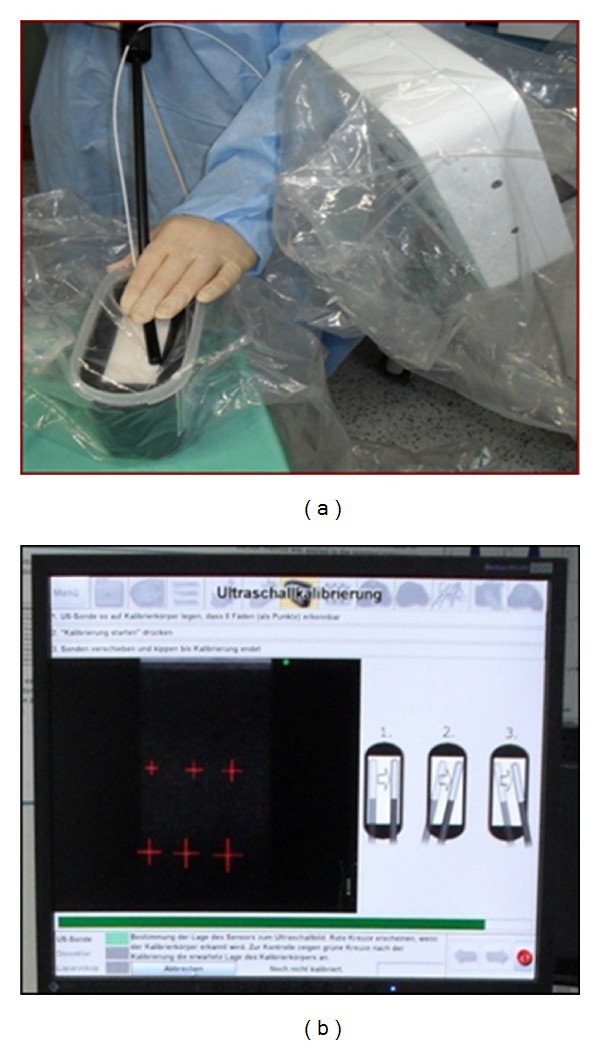
Calibration. (a) Calibration of the ultrasound device by placing it onto the calibrating phantom, (b) calibration process by registered strands in the calibrating phantom.

The entire system has to be calibrated to the individual setting before the beginning of the surgical procedure (Figure 3). This involves the laparoscopic instruments (camera, dissector, and ultrasound device). Calibration takes place under sterile conditions. The exact calibration of the dissecting instrument is essential, for which a phantom is used by applying pivotal shift and rotation. For calibration of the camera the field generator is positioned in front of the camera which for this step is mounted in a certain position (Figure 4). The camera is then enlined with the squares which are turned after each registration by the system. The process of turning and subsequent registration is repeated five times. Calibration of the ultrasound device is carried out in a similar manner by positioning the system on a calibration phantom. The ultrasound B-Mode view has to show six strands with a calibrated position in the phantom (Figures 5(a) and 5(b)). The defined positions are then registered by the software. Then, the position of the mounted tracking adapter at the tip of the laparoscopic ultrasound probe is known and the ultrasound probe may be used for further operative navigation. 

### 2.5. Intraoperative Liver Registration

**Figure 6:**
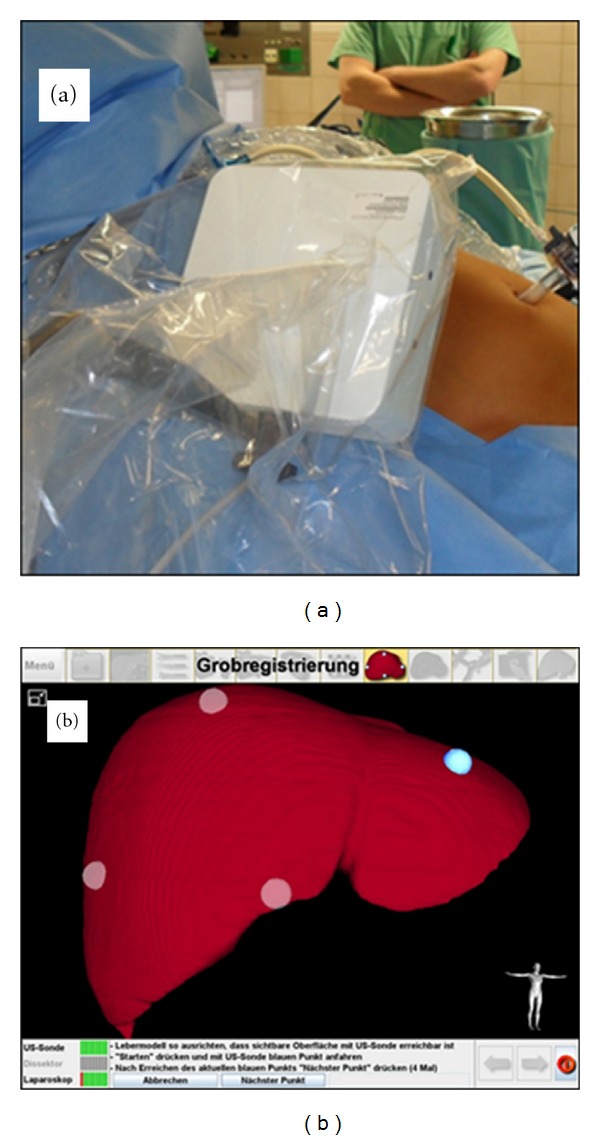
Intraoperative liver registration. (a) Positioning of the field generator beside the patient, (b) liver registration using four defined spots. The landmark on the left lobe is already activated.

Following the calibration of the laparoscopic instruments a pre-registration of the patient's liver is performed. The aim of this step is a rapid and crude measurement of the liver. For this, the field generator is positioned next to the right upper abdomen of the patient. Then, four defined spots on the liver surface (cranial, caudal, left, and right) are focussed by using the ultrasound device (Figures 6(a) and 6(b)). The detailed registration of the entire liver surface is achieved by scanning the whole organ with the ultrasound device. This implies the scan and imaging of the tumor and critical vascular structures including the Vena cava and the portal vein.

## 3. Results

### 3.1. Intraoperative Course

**Figure 7:**
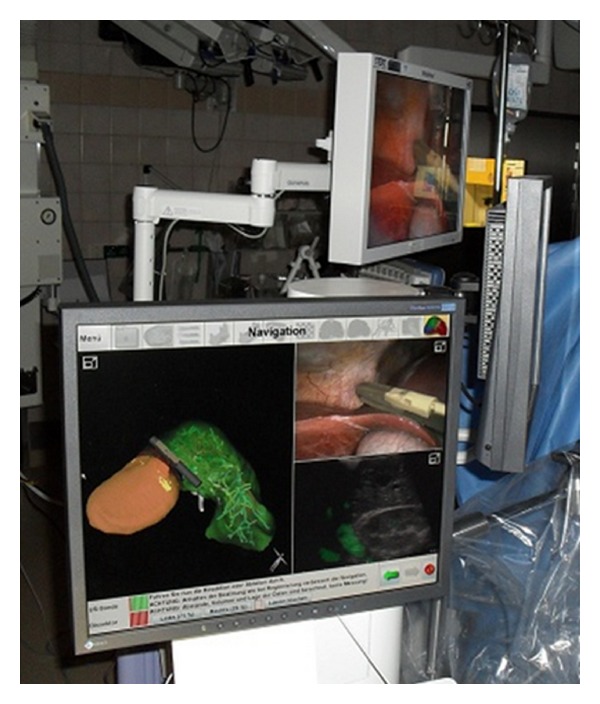
LapAssistent placed intraoperatively on the right patient side; simultaneous laparoscopic and ultrasound scenes are described above.

The Patient was kept in *Y*-position. The sterile navigation system LapAssistent was placed on the right side of the patient (Figure 7). First of all, minilaparotomy, trocar placement, creation of the pneumoperitoneum, positioning of the camera, and diagnostic laparoscopy were performed to exclude extrahepatic pathologies. Then, the laparoscopic instruments with real-time ultrasound B-Mode screen and real-time laparoscopic video screen were connected to the LapAssistent. The navigated ultrasound B-Mode image was introduced into the preoperative planning scene. The planned resection plane was adjusted by ultrasound. The definition of the resection plane of the liver capsula was marked with electro-coagulation. After mobilization, the tumor was resected by laparoscopic stapling devices. Using a retrieval bag and via minilaparotomy the tumor was removed. The overall operative time was 141 min, including 18 minutes for system setup and calibration process. The technical and sterile use of the system was feasible. All image data were transferred and visualized intraoperatively. The proposed resection plain was controlled intraoperatively with ultrasound device, and it was found that this corresponded closely to the resection which would have been chosen on the basis of macroscopic findings alone. Changes in the patient's position from the normal to, for example, anti-Trendelenburg position led to gravity displacement of the liver with navigation inaccuracy and repeated registration of the liver surface was necessary in these situations. An influence of ventilation could not be detected.

### 3.2. Postoperative Course

**Figure 8:**
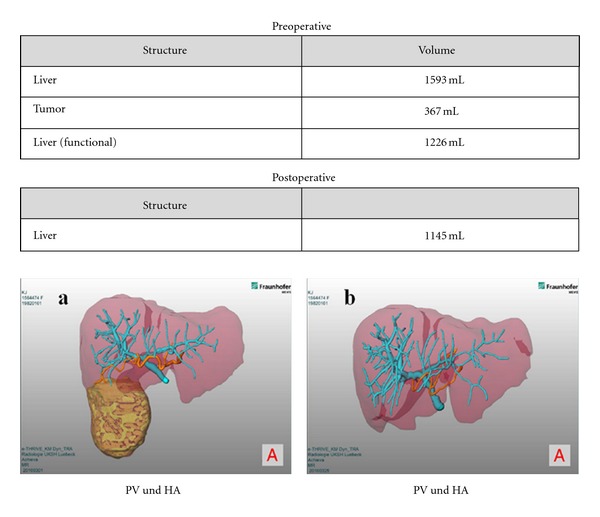
Comparison of pre- and postoperative liver volume. (a) Preoperative 3D presentation of liver with portal venous and arterial vessels and tumor location. (b) Postoperative 3D presentation of liver with portal venous and arterial vessels.

To control the resection outcome we accomplished MRI on day 8 after surgery (Figure 8). The residual liver volume was 1145 mL and therefore 81 mL less than determined by the preoperative planning data. Pathological evaluation revealed a tumor-free resection margin of a liver cell adenoma. On the sixth postoperative day the patient was discharged without any complications. One-year follow-up revealed an excellent clinical course. A recurrent MRI liver scan showed no signs of new liver pathologies.

## 4. Discussion

Interventional navigation systems have been widely used in neurosurgery, otolaryngology, and orthopedic surgery to improve intraoperative orientation and to increase accuracy of tumor localization or bone resection. This was possible due to the rapid progress in new imaging modalities in radiology together with the continuous improvement in high performance computing technology [16–18]. We here present a novel tool for navigated laparoscopic liver surgery that enables the intraoperative assistance of preoperative 3D-planning data. The positive impact of preoperative 3D-planning data in open liver surgery was reported by Lang et al. for major liver resections [19]. Also, Beller et al. reported the combined use of optoelectronic and electromagnetic navigation in open liver resection [20]. They presented 5 patients and were able to overcome the obstacle of soft tissue deformation during the intervention with preliminarily acceptable results. Our own first clinical results show that the system successfully supports the surgeon during an intervention. In its current state, the LapAssistent provides a unique interface integrating visualization, alignment of planning data to the intraoperative situation by rigid landmark, and liver surface registration by the use of the electromagnetic tracking system. The advantages of electromagnetic tracking in contrast to optical tracking methods in laparoscopic procedures have been described [21, 22]. As a major advantage, the navigation information from a tracker placed at the tip of the instrument, in our case the laparoscopic ultrasound system, reduces the error in soft tissue navigation. So, angulation of the tip of the ultrasound probe does not cause measurement errors. The accuracy of navigation based on 3D ultrasound is important if the technology should represent an improvement of instruments used in existing laparoscopic procedures. But many parameters may affect the accuracy of navigated laparoscopic ultrasound. Except for movement of organs, the most important factor influencing the accuracy may be the nature of the tracking system [22]. For this reason, we do not overlap the preoperative 3D-CT-Data to the operation field, but we overlap the position and B-Mode view of the laparoscopic ultrasound into the preoperative data. So, to our knowledge, we exclude any registration errors from the operating field but still receive the guidance by the LapAssistent system. Although navigation technology is not frequently used in laparoscopy at present, in a Norwegian survey the surgeons were positive toward an increased use of laparoscopic ultrasound (LUS) and navigation technology combined with LUS. According to the surveyed surgeons, LUS is a promising tool that increases surgical safety and improves tumor resection [23]. Although automatic non linear registration has not been implemented yet, our system allows reasonable intra-abdominal navigation of laparoscopic interventions. From our experience we found that the human-computer interaction is convenient for intraoperative application. Nevertheless, for successful handling the surgeon needs profound training. The major problem yet is poor compounding of 2D-US slices such that we have no 3D-US volume of sufficient quality available. The technical and sterile use of the system was feasible. The evaluation of the resected material attested a tumor-free resection margin. Also, the postoperative MRI scan demonstrated a sufficient resection. The operating time was 141 min. A literature review covering series from 1998 to 2009 on laparoscopic liver resection reported a mean operative time of 179 min [7]. Here, however, the beginnings of laparoscopic liver surgery and extensive liver resections are to be considered. Our first clinical application showed the safe use of a laparoscopic navigation system for intraoperative planning transmission in laparoscopic liver surgery. For the future, we must determine how these early experiences are comparable in a larger patient population. Therefore, the German prospective multicentre ProNaviC I trial was conducted and launched (Prospective multicenter pilot study of perioperative evaluation of surgical navigation assistance systems for liver tumors).
